# Kinetics of foot-and-mouth disease antibody response in cattle vaccinated with FMD-HS-BQ combined vaccine under field condition

**DOI:** 10.3389/fmicb.2026.1800886

**Published:** 2026-05-22

**Authors:** Rajeev Ranjan, Himanshu R. Joshi, Vinay Pandey, Jitendra Kumar Biswal, Samarendra Das, Bighneswar Barik, Jajati Keshari Mohapatra, Smrutirekha Mallick, Rabindra Prasad Singh

**Affiliations:** 1 ICAR-National Institute on Foot and Mouth Disease, Bhubaneswar, Odisha, India; 2 Wildlife Conservation Trust, Mumbai, India

**Keywords:** cattle, FMD-HS-BQ combined vaccine, foot-and-mouth disease, immune response, SPCE

## Abstract

Foot-and-mouth disease (FMD), Hemorrhagic septicemia (HS), and Black quarter (BQ) are economically important diseases of livestock and wildlife species worldwide, with vaccination serving as the primary control strategy. This study examined the development and duration of FMD antibody responses in cattle administered a trivalent FMD-HS-BQ vaccine under field conditions. Thirty cattle were allocated to three groups: Group 1 consisted of naïve calves (6 receiving a booster vaccination and 6 without a booster); Group 2 included adult cattle with an unknown vaccination history (6 with a booster, 6 without a booster); and Group 3 served as unvaccinated controls (6 animals). Blood samples were collected on days 0, 28, 120, 240, and 365 following initial vaccination, and anti-FMDV antibody levels were measured using solid-phase competitive ELISA. The combined vaccine stimulated anti-structural protein antibody production against FMDV in cattle, with antibodies remaining detectable through day 365 and beyond in animals receiving booster vaccination. Conversely, antibody levels in non-boosted animals started declining after day 120. Virus neutralization testing confirmed the presence of protective neutralizing antibodies on day 365 in cattle that received booster doses. These findings suggest that regular administration of the FMD-HS-BQ combined vaccine at wildlife-livestock interfaces could provide effective FMD protection while offering economic advantages through reduced vaccination costs, labor requirements, and stress on both animals and handlers.

## 1 Introduction

Foot-and-mouth disease (FMD), Hemorrhagic septicemia (HS), and Black quarter (BQ) are three important infectious diseases of livestock and wildlife species globally (Alexandersen et al., 2003; Echenique et al., 2018; Shome et al., 2019). FMD causes significant economic losses in the livestock sector globally. In India itself, there has been a direct loss of up to USD 3159 million (INR 221,110 million) annually due to FMD (Govindaraj et al., 2021). FMD is a contagious viral disease caused by *Aphthovirus*, a member of the family *Picornaviridae* (Belsham, 1993). FMD virus (FMDV) affects cloven-hoofed domestic animals such as cattle, buffalo, sheep, goats, and pigs, as well as more than 70 wildlife species (Alexandersen et al., 2003). FMDV-infected animals showed clinical signs such as high fever, hyper salivation, and lesions on the tongue, feet, and teats. After the clinical phase, a subset of FMDV persists at the site of predilection in the host (Hayer et al., 2018; Ranjan et al., 2025a). At present, FMD virus (FMDV) serotypes O, A, Asia1, SAT-1, SAT-2, and SAT-3 are in global circulation. In India, FMDV serotypes O, A, and Asia 1 are in circulation, with the maximum number of outbreaks due to serotype O (Subramaniam et al., 2022).

FMD is a viral disease with no effective treatment; only symptomatic treatment can be provided to the affected animals. Moreover, treatment was not feasible for wildlife. Therefore, in an endemic setting, vaccination is the only available approach to prevent FMD. Due to regular vaccination, a few countries are very close to achieving FMD-free status, but some are in the early stages of FMD control, and a few are ready to implement FMD control programs using regular vaccination policies to control and eliminate FMD.

Depending on the epidemiological situation, combined vaccines in endemic settings frequently use two or more different antigens. The use of combined vaccines is very common in humans and canines, *viz.*, human hexavalent (6-in-1), dogs DLPP/7-in-1, and others. However, in the livestock sector, the use of a combined vaccine is very limited. A combined vaccine formulation would be extremely beneficial to farmers and the livestock sector by avoiding unnecessary financial burdens and logistics expenses, and reducing stress on animals from frequent vaccinations for multiple diseases. Cost–benefit analyses are critical for vaccinations in the livestock industry (Çokçalışkan et al., 2019). Furthermore, it could be helpful to reduce the costs of vaccine development, vaccination, and manpower for effective delivery by administering two or more immunogens in one injection (Palanisamy et al., 1992). A single-injection policy may also reduce the risk of local reactions from multiple injections.

Previous reports have revealed that the development of a combined vaccine based on FMD has shown potential for success (Chhabra et al., 2004; Reddy et al., 2004; Altaf et al., 2012; Prasad et al., 2019; Muenthaisong et al., 2021; Sareyyüpoğlu et al., 2022; Yaser et al., 2023). The vaccine currently used in India under the National Animal Disease Control Program (NADCP) operated by the Government of India against FMD is a trivalent inactivated vaccine containing FMDV serotypes O, A, and Asia 1. Only one combo vaccine against FMD, HS, and BQ is available in the country; this combined vaccine may give protection against all three pathogens, but there is a dearth of literature dealing with the determination of the duration of the persistence of protective antibodies against FMDV in cattle after administration of the FMD-HS-BQ combined vaccine (Mishra et al., 2025).

The wildlife-livestock interface is a place where livestock like cattle, buffalo, sheep, and goats are present and interact with wildlife herbivores, and may play a crucial role in the epidemiology of FMD transmission. It has high densities of spotted deer (*Axis axis*) and moderate densities of other ungulate species such as sambar, nilgai, wild boar, chinkara, and barking deer (Jhala et al., 2020). It has also not been possible to vaccinate each animal with a separate vaccine for different diseases, as this would impose unnecessary financial burdens, increase logistical costs, and cause undue stress on animals due to frequent vaccinations for multiple diseases. The FMD-HS-BQ combined vaccine could be used regularly at WLI to protect against FMD, reduce vaccination costs and labor, and reduce the biotic stress on animals and animal keepers. Therefore, the present study aimed to investigate the kinetics of FMD antibody (Ab) response in cattle vaccinated with FMD-HS-BQ combined vaccine under field conditions at the wildlife-livestock interface (WLI), which could generate information on the efficacy of such vaccine in creating an immune belt and in preventing the transmission of FMDV infection either from wildlife to domesticated livestock or vice versa.

## 2 Materials and methods

### 2.1 Study area and animals

The present study was carried out in non-descript indigenous cattle at the Wildlife-Livestock Interface (WLI) of Sanjay Tiger Reserve (STR) and Bandhavgarh Tiger Reserve (BTR), Madhya Pradesh, India. Non-descript Indigenous cattle, buffalo, goats, and sheep are being reared in a free-range system in this area. Before vaccination, all animals used in this study were dewormed using a suitable anthelmintic suggested by the local veterinarian. These animals were also negative for diseases such as brucellosis, tuberculosis, and Johne’s disease. Thirty animals were divided into three groups (Gr), namely Gr1, Gr2, and Gr3. Gr1 consisted of 12 calves; calves included in the present study are negative for 3AB3 NSP Abs against FMD virus and genome for FMD virus in oropharyngeal fluid (OPF) for FMDV persistence as described earlier (Ranjan et al., 2018) and further redistributed into the booster group (*n* = 06) and non-booster group (*n* = 06). Gr2 consisted of 12 adult cattle (without any history, vaccinated or unvaccinated) and were redistributed into the booster group (*n* = 06) and non-booster group (*n* = 06). Gr3 was kept as unvaccinated controls (*n* = 06) as depicted in Table 1. In the present study, Gr1, Gr2, and Gr3 were taken to generate a longitudinal dataset to assess the persistence of protective Ab against FMD.

**Table 1:** Grouping (number) of animals used in the longitudinal study on the duration of persistence of protective antibodies (titer ≥ log_10_ 1.65) against FMDV in cattle after administration of FMD-HS-BQ combined vaccine at the wildlife-livestock interface (WLI).

Group of animals	Booster group	Non-booster group
Group 1 (Calf naïve)	06	06
Group 2 (Adult cattle)	06	06
Group 3 (Unvaccinated cattle control)	06	

### 2.2 Vaccine and vaccination

**Figure 1:**
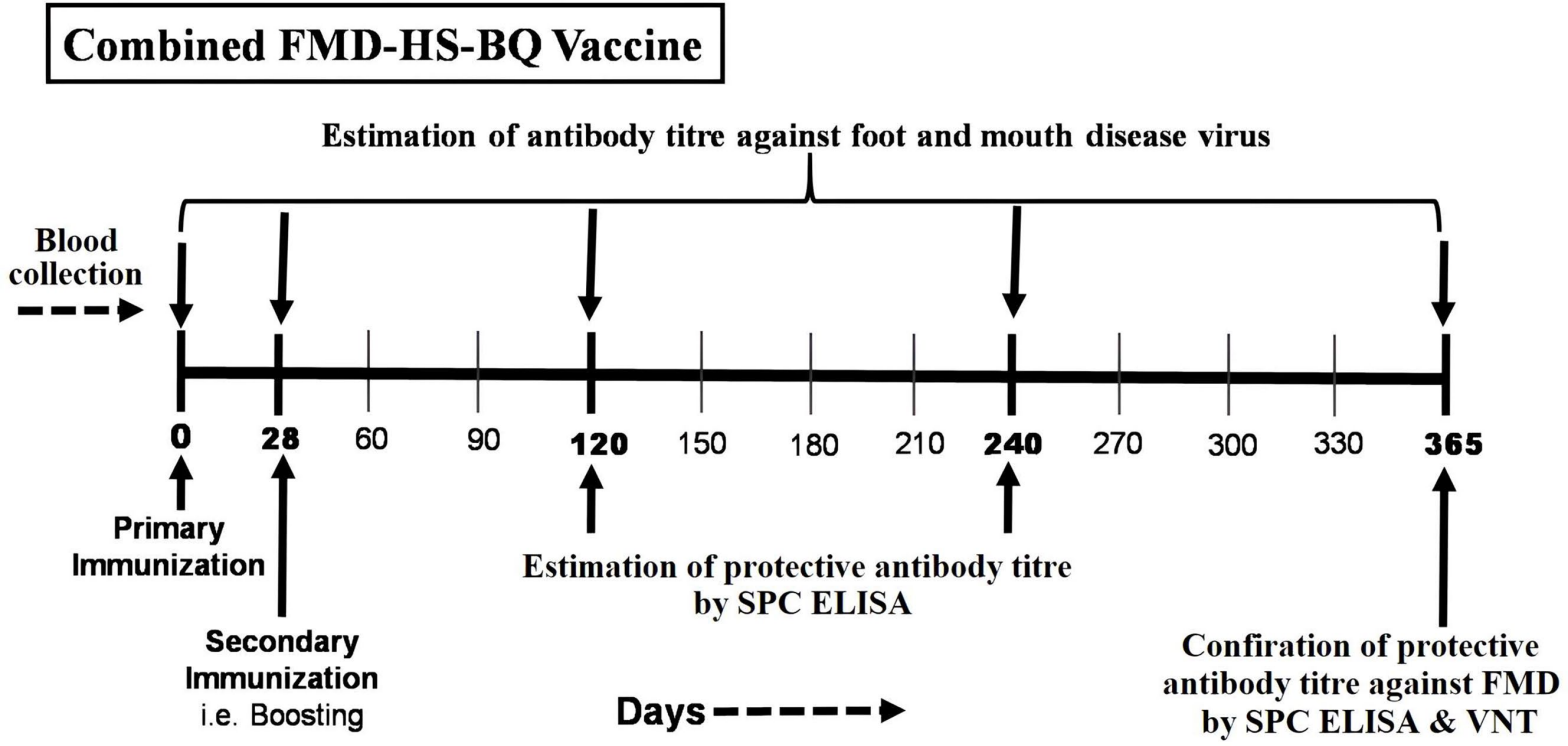
Vaccination regimen, sample collection schedule, and evaluation of FMDV-protective antibody titers in cattle vaccinated with FMD-HS-BQ combined vaccine.

Commercially available combined vaccine, Raksha Triovac (Foot and mouth disease + Hemorrhagic septicemia + Black Quarter oil adjuvant vaccine), Batch No.-01RTN00322, was used in the present study. The vaccine used in this study is a Combined Oil-Adjuvanted vaccine (FMD, HS, and BQ) containing inactivated *Pasteurella multocida*, *Clostridium chauvoei*, and FMDV serotypes O, A, and Asia 1 antigens. FMDV vaccine strains used in the commercial vaccine are FMD serotypes O IND R2/1975, A IND 40/2000, and Asia1 IND 63/1972. The in-use vaccine strains perfectly match the circulating viruses antigenically, as revealed in the 2D-VNT vaccine regularly conducted at ICAR-NIFMD, National Referral Laboratory for FMD in India (ICAR-NIFMD, 2022). Non-descript Indigenous cattle received 3 mL, deep intramuscular FMD-HS-BQ combined vaccine in the neck region using an 18G needle as per the manufacturer’s (Indian Immunologicals Limited) instructions. The first dose of vaccine was administered in Gr1 &Gr2 on the 0th day (D0, vaccination day) in the month of December, followed by the administration of the booster dose at 28 days post-vaccination (DPV), and samples were collected for 1 year as depicted in Figure 1.

### 2.3 Sample collection

Blood samples were collected at 0, 28, 120, 240, and 365 DPV to determine the Ab titer against FMDV. As per the Committee for Control and Supervision of Experiments on Animals (CCSEA) guideline, whole blood samples (4 mL) were collected by jugular venipuncture from individual animals in an eVac tube with gel and clot activator (Peerless Biotech Pvt. Ltd.) as described in the institutional animal ethics committee (IAEC) protocol (P13-03/2023). Serum was separated from whole blood samples by centrifugation at 3,000 rpm for 20 min, transported to the testing laboratory, ICAR-National Institute on Foot and Mouth Disease (NIFMD) under a cold chain, and then preserved at −40 °C till further use and thawed at 37 °C before use.

### 2.4 Estimation of antibody titer against FMD virus

#### 2.4.1 Estimation of anti-structural protein (SP) antibody titer against FMDV by solid phase competitive ELISA (SPCE)

FMDV vaccine strains used in the commercial vaccine are FMD serotypes O IND R2/1975, A IND 40/2000, and Asia1 IND 63/1972. Estimation of anti-SPS Abs titer against the FMDV serotypes O, A, and Asia1 was performed using SPCE as described by Pannu et al. (2025). The sensitivity of this test was 90%, whereas the specificity was 100%. In brief, SPCE was performed, and the optical density (OD) was measured at 492 nm with a reference at 620 nm using an ELISA reader (Thermo Fisher, Multiscan). When antigen and background controls showed OD values between 0.8 and 0.1, the test was considered valid. The percent of inhibition (PI) was calculated using the following formulas:

$$\text{Percent of Inhibition}\;(\mathrm{PI})=100-\frac{\begin{array}{l} \text{Mean}\;\mathrm{OD}\;\text{of Sample}\\ -\text{Mean}\;\mathrm{OD}\;\text{of}\;\mathrm{Cc} \end{array}}{\begin{array}{l} \text{Mean}\;\mathrm{OD}\;\text{of}\;\mathrm{Co}\\ -\text{Mean}\;\mathrm{OD}\;\text{of}\;\mathrm{Cc} \end{array}}\times 100$$

Where, Cc = Conjugate control; Co = Antigen control.

The log_10_ value of the test sample dilution showing 35% inhibition was taken as the Ab titer for the given samples. Test samples showing log_10_Ab titer ≥1.65 were considered as protective Ab titer. SPCE is applied for the estimation of protective Ab titer against FMD routinely under the National FMD Control Program in India, as per the WOAH guideline for post-vaccination seromonitoring (PVM), and before implementation, it has been properly optimized and validated with VNT for deciding the threshold in SPCELISA of protective antibody titer against FMDV (ICAR-NIFMD, 2022).

#### 2.4.2 Estimation of neutralizing antibody titer in serum against FMDV by virus neutralization test

Virus neutralization test (VNT) is considered the gold-standard test for the estimation of the neutralizing Ab titer against FMDV; therefore, VNT was performed on serum samples collected on the 365th DPV to confirm the presence of FMD-neutralizing Ab and assess the duration of persistence of protective Ab (Biswal et al., 2022). In brief, serum samples were collected and heat-inactivated at 56 °C for 30 min, and VNT was performed using homologous FMDV. Fifty μL of two-fold serially diluted serum samples were added to a 96-well cell culture plate, followed by the addition of 50 μL of 100 TCID_50_ of the virus to each well, and the plate was incubated at 37 °C for 1 h. After incubation, approximately 5.0 × 10^4^ BHK-21 cells were added to each well to assess residual viral infectivity. Healthy cells and FMDV control wells were kept in every assay. The cell-culture plates were incubated at 37 °C for 48 h, then the neutralizing Ab against FMDV was calculated as the reciprocal of the highest serum dilution that neutralized 100 TCID_50_ of the virus in 50% of the wells.

### 2.5 Proportion value calculation

The original Ab titer results were recorded in threshold format (i.e., either greater than or less than a cut-off value, rather than continuous exact values). In such cases, representing the data as proportions of animals above or below the threshold provides a more interpretable and population-relevant perspective, especially for evaluating vaccine efficacy under field conditions. The rationale for converting the test results into proportions is that this continuous, normalized form is ideal for various statistical analyses, including model fitting. Therefore, proportion values were computed by dividing the number of animals with positive titers at each time point by the total number of animals tested in the respective group at that time point.


$$\text{Proportion positive value}=\frac{\begin{array}{l} \mathrm{No}.\text{of animals showing}\;\\ \log10\;\mathrm{Ab}\;\text{titer}\geq 1.65 \end{array}}{\begin{array}{l} \text{Total number of}\\ \;\text{animal tested} \end{array}}$$

Consider clarifying with an example (e.g., “if 2 out of 6 animals showed positive results, the proportion was reported as 0.33”) for better comprehension. This proportion value was converted into a percent by multiplying by 100.

### 2.6 Statistical analysis

The statistical significance of the duration of persistence of protective Ab in cattle vaccinated with the FMD-HS-BQ combined vaccine under field conditions at WLI was carried out using Fisher’s exact test and a polynomial regression model. These methods were applied for comparison of anti-SP and Ab titer across different cattle groups [Calf-naïve (Booster, B), calf-naïve (non-booster, NB), adult-cattle (Booster, B), adult-cattle (non-booster, NB), and control] at different time points (0, 28, 120, 240, and 365 DPV). *Post hoc* power analysis confirmed that the study design provided adequate statistical power to detect large booster-associated differences in antibody levels, even with a subgroup size of *n* = 6.

#### 2.6.1 Fisher’s exact test

Fisher’s exact test was carried out for pairwise comparisons at each time point. This largely accounted for the differences in proportions of positive Ab titer between groups over time. Fisher’s exact test is proper when the sample sizes are small. It has been used to compare binary outcomes, that is, positive vs. negative Ab titers, across time points between groups. Here, the positive and negative titer groups are defined as animals having log_10_ titers greater than and less than the defined threshold of log_10_ 1.65, respectively.

The 2 × 2 contingency table for the test is given as:

**Table:** 

	Positive	Negative
Group A	a	b
Group B	c	d

The formula to calculate the *p*-value is given as:


$$p=\frac{\left(\begin{array}{c} a+b\\ a \end{array})(\begin{array}{c} c+d\\ c \end{array}\right)}{\left(\begin{array}{c} n\\ a+c \end{array}\right)}$$

Where

- *a*, *b*, *c*, and *d* represent the cell frequencies
- *n* = *a* + *b* + *c* + *d* is the total number of observations
-
$\left(\begin{array}{c} n\\ k \end{array}\right)$ is the binomial coefficient, given as:

$$(\begin{array}{c} n\\ k \end{array})=\frac{n!}{k!(n-k)!}$$

In simple words, *a* and *b* are the number of positives and negatives in Group A, whereas *c* and *d* are the number of positives and negatives in Group B.

Null Hypothesis (H₀): $P_{1}=P_{2}$ , There is no significant difference in the proportion of positive cases. Alternative Hypothesis (H₁): $\;P_{1}\neq P_{2}$ , There is a significant difference in the proportion of positive cases. If *p*-value ≤ α (0.05): Reject the null hypothesis (H₀). This suggests a statistically significant difference between the proportions of positive cases. If *p*-value > α (0.05): Fail to reject the null hypothesis (H₀). This suggests that there is no statistically significant difference between the proportions of positive cases across the two groups.

#### 2.6.2 Polynomial regression model

To fit the curve of anti-SP antibody titer against different serotypes of FMDV versus time (in DPV) and to make valid predictions at subsequent stages, a second-degree polynomial regression model with orthogonal polynomial terms was used. This model catches the non-linear characteristics of the antibody response, including the initial increase after vaccination and subsequent drop, typical of immune kinetics.

The general polynomial regression model is given as


$$y_{i}=\beta_{0}+\beta_{1}z_{i1}+\beta_{2}z_{i2}+\varepsilon_{i}$$

Where.

$z_{i1}$ and $z_{i2}$ are the first and second degree orthogonal polynomial transformations of $x_{i}$ ;

$\beta_{0}$ , $\beta_{1}$ , and $\beta_{2}$ are the estimated coefficients; $\varepsilon_{i}$ : random noise with N (0, 1).

This regression-derived methodology allowed for smoothed estimation of antibody response trends, enabling reliable comparisons between groups based on peak response level, rate of decline, and duration of elevated titer. The model, in accommodating time-dependent non-linear trends, facilitates a more accurate assessment of vaccine efficacy and long-term duration of immunity.

## 3 Results

**Figure 2:**
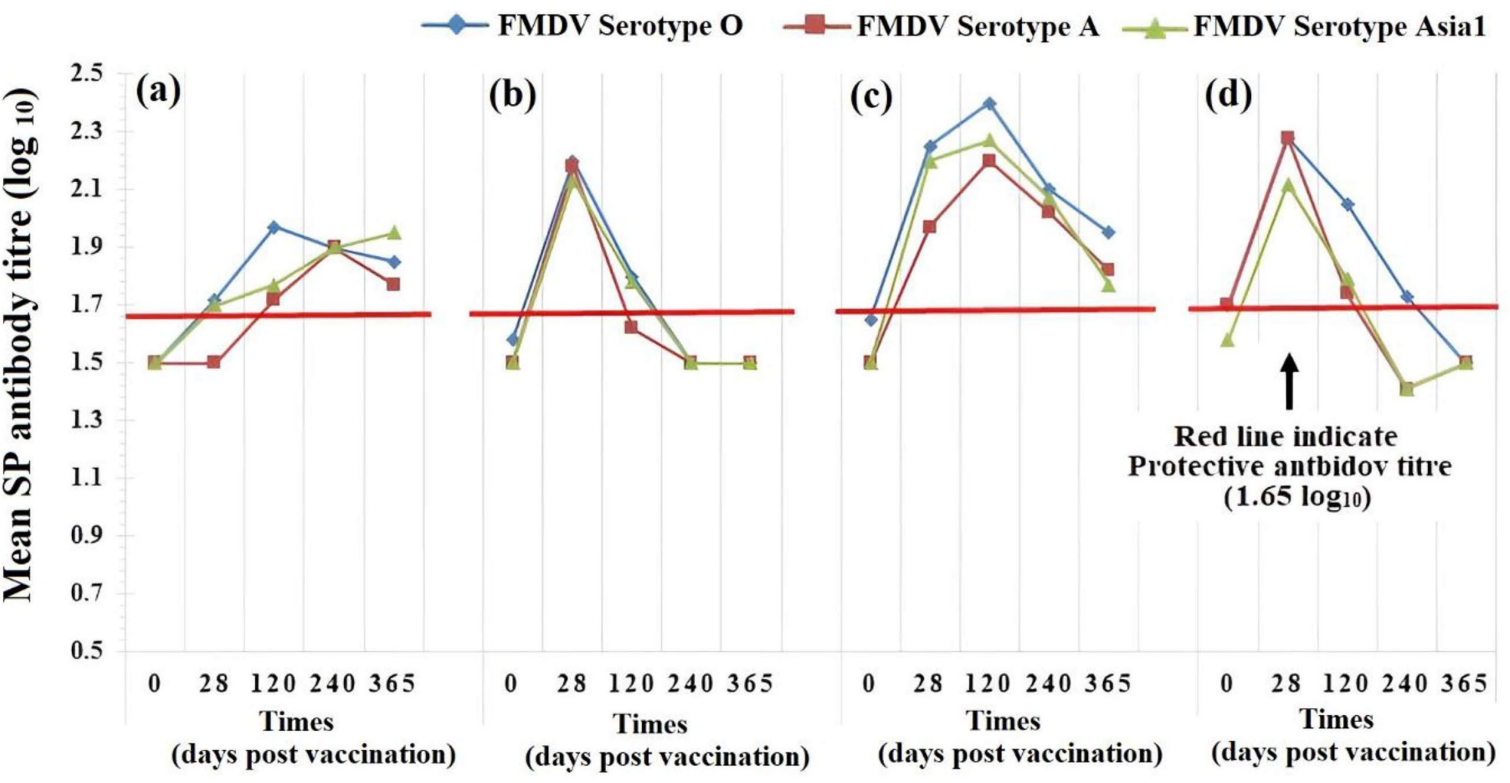
Comparison of the geometric mean of anti-structural protein (SP) antibody titers (log_10_ antibody titer) against foot-and-mouth disease virus (FMDV) up to 365 days post vaccination (DPV) for different groups of animals [**(a)** calf-naïve (B, booster), **(b)** calf-naïve (NB, non-booster), **(c)** adult-cattle (B, booster), and **(d)** adult-cattle (NB, non-booster)] immunized with FMD-HS-BQ combined vaccine.

**Figure 3:**
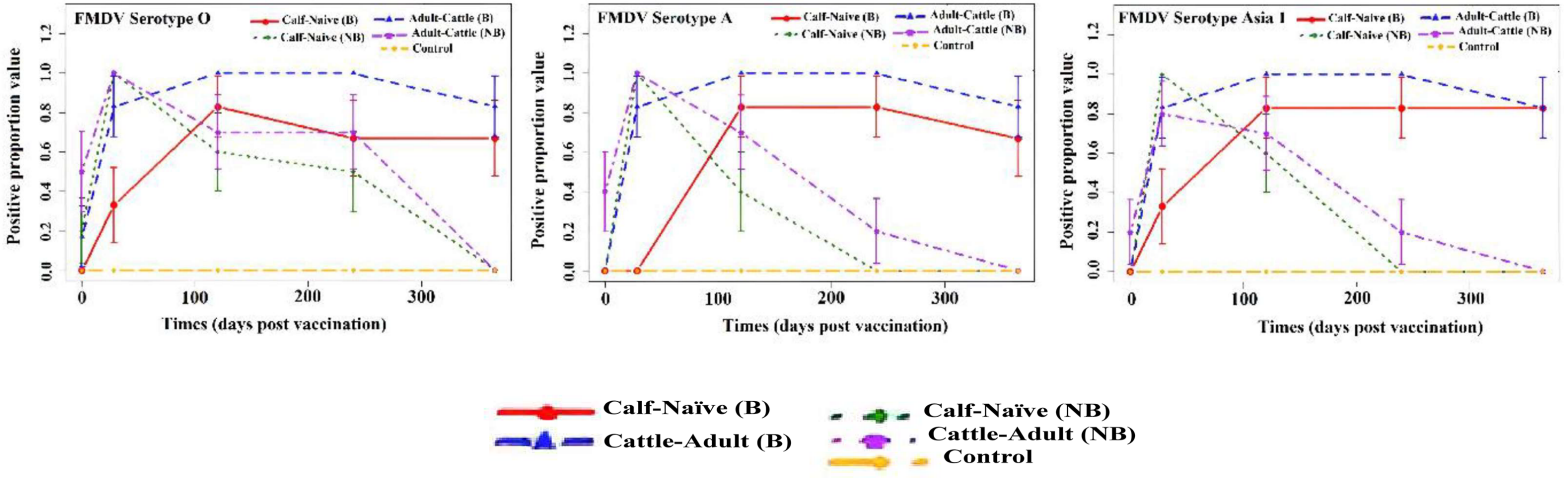
Comparison of positive proportion value with standard error of the mean (shown as bars) at each time point, different groups of animals [calf-naïve (B, booster), calf-naïve (NB, non-booster), adult-cattle (B, booster), adult-cattle (NB, non-booster), and control] against foot-and-mouth disease virus (FMDV) serotype O, A, and Asia1 after immunization with FMD-HS-BQ combined vaccine in cattle. Positive proportion values are the number of animals with protective antibody titers (≥1.65 log_10_) divided by the total number of animals tested.

**Figure 4:**
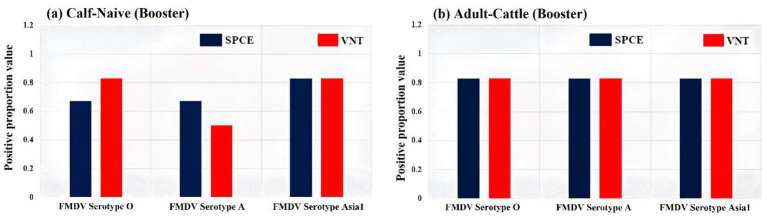
Comparison of positive proportion value [the number of animals with protective antibody titer (≥1.65 log₁₀) divided by the total number tested] of anti-structural protein (SP) antibodies titer by virus neutralization tests (VNT) and solid phase competitive ELISA (SPCE) at 365 days post vaccination (DPV) against foot-and-mouth disease virus (serotype O, A, and Asia 1) for **(a)** Calf naïve (booster) and **(b)** for adult-cattle (booster) immunized with FMD-HS-BQ combined vaccine.

**Figure 5:**
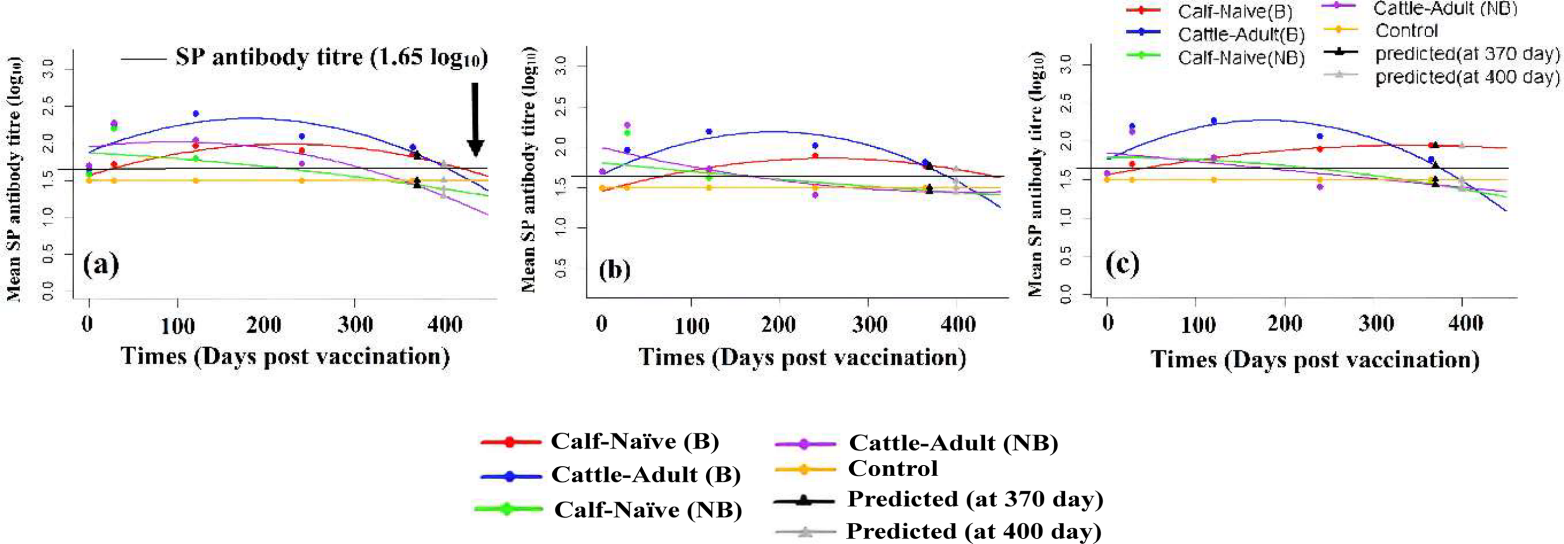
Plotting of fitted polynomial regression models for mean anti-structural protein (SP) antibody titer (log_10_) and predicting for 370 and 400 days post-vaccination (DPV) against foot and mouth disease viruses: **(a)** Serotype O, **(b)** serotype A, and **(c)** serotype Asia 1 in different groups of animals [calf-naïve (B, booster), calf-naïve (NB, non-booster), adult-cattle (B, booster), adult-cattle (NB, non-booster), and control] immunized with FMD-HS-BQ combined vaccine.

In group 1, 12 FMDV-negative naïve calves were included in this study. To determine their FMDV-negative status, the serum samples were analyzed by 3AB3-NSP ELISA (Mohapatra et al., 2011), and oropharyngeal fluid samples were analyzed by RT-PCR (Ranjan et al., 2018) to rule out the presence of either the FMDV-specific non-structural protein antibody or the viral genome, both of which are indicators of virus circulation. After deep intramuscular inoculation of 3 mL of FMD-HS-BQ combined vaccine on 0 DPV, followed by 2nd inoculation, i.e., booster dose on 28 DPV in Gr1 (calves) and Gr2 (cattle), no adverse reactions were observed. Longitudinal humoral immune response against FMDV in cattle after inoculation of FMD-HS-BQ combined vaccine was studied (Figures 2–5; Supplementary Tables S1–S4). Blood samples were collected from animals at 0, 28, 120, 240, and 365 DPV and tested for anti-SP Abs titers against FMDV.

A post-hoc power analysis to quantify detectable effect sizes with subgroup n = 6 and α = 0.05. Comparisons involving booster-vaccinated naïve calves consistently exhibited large effect sizes and strong power estimates, particularly for serotype Asia1 (Cohen’s d = 1.857–1.954; Power = 0.825–0.861) and serotype O (d = 1.404–1.607; Power = 0.593–0.709). The analysis indicates that the design has good power to identify large booster effects, but insufficient power to detect small incremental differences or subtle antigenic interactions. A detail of the *post hoc* analysis has now been attached and presented in Supplementary Table S4.

### 3.1 Estimation of antibody titer against the anti-structural protein (SP) of FMDV in cattle

The graph illustrates the comparison of anti-SP Abs titer against the FMDV (serotype O, A, and Asia 1) geometric mean and positive proportions over time across three groups (Gr 1, Gr 2, and Gr 3) consisting of five categories of animals: calf-naive (B), adult-cattle (B), calf naive (NB), cattle-adult (NB) immunized with FMD-HS-BQ combined vaccine, and unvaccinated control is depicted in Figures 2–5 and in Supplementary Tables S1–S3.

Geometric mean of actual anti-SP Abs titer against FMDV of different serotypes over time for different groups of animals immunized with FMD-HS-BQ combined vaccine depicted in Figure 2. The calf-naive (B) group has a gradual increase, reaching its peak at 120 DPV for serotype O and at 240 DPV for serotype A and Asia 1. The calf-naive (NB) group demonstrates an early peak at 28 DPV, followed by a consistent decline in protective antibody titer, indicating a diminishing response over time. Similarly, the adult-cattle (B) group shows the highest peak at 120 DPV and maintains a protective antibody titer through 365 DPV. Like calf naïve (NB) and adult-cattle (NB) groups, which also exhibit a high immune response, peaking at 28 DPV, they maintained a protective antibody titer through 120 DPV for all three serotypes. But protective Ab titers for serotype A and Asia 1 started declining by 120, and for serotype O by 240 DPV. Overall, the immune response in the calf-naïve group was lower than that of the adult groups. Similarly, the duration of persistence of protective Abs was longer in the booster group than in the non-booster group.

Anti-SP Ab titer against FMDV serotype O virus positive proportion over time for different groups of animals immunized with FMD-HS-BQ combined vaccine depicted in Figure 3. The adult-cattle (B) group stands out with the highest peak at 28 DPV. It maintains a high proportion close to 1.0 until 240 DPV, after which it shows a slight decline in protective antibody titer by 365 DPV, making it the best performer in terms of maintaining a high positive proportion over time (Table 2). The adult-cattle (NB) group also exhibits a strong initial response, peaking at 28 DPV and maintaining a relatively high proportion until 240 DPV, but it shows a more significant decline by 365 DPV. The calf-naive (B) group shows a gradual increase, peaking at 120 DPV, then stabilizing and maintaining a constant proportion through 365 DPV, suggesting a sustained response, albeit at a lower level compared to the adult groups. The calf-naive (NB) group demonstrates an early peak at 28 DPV, followed by a consistent decline, indicating a diminishing response over time. The Control group (Gr3) remains at zero throughout.

**Table 2:** *P*-value from Fisher’s exact test for FMD virus serotypes O, A, and Asia 1 between different times for different groups.

	FMDV serotype “O”					FMDV serotype “A”					FMDV serotype “Asia 1”				
Days	0	28	120	240	365	0	28	120	240	365	0	28	120	240	365
	Calf-naïve (Booster)														
0	–	NS	0.015	0.05	0.05	–	NS	0.015	0.015	0.05	–	NS	0.015	0.015	0.015
28	NS	–	NS	NS	NS	NS	–	0.015	0.015	0.05	NS	–	0.24	0.24	0.24
120	0.015	NS	–	NS	NS	0.015	0.015	–	NS	NS	0.015	NS	–	NS	NS
240	0.05	NS	NS	–	NS	0.015	0.015	NS	–	NS	0.015	NS	NS	–	NS
365	0.05	NS	NS	NS	–	0.05	0.05	NS	NS	–	0.015	NS	NS	NS	–
	Calf-naïve (Non-booster)														
0	–	0.015	NS	NS	NS	–	0.002	NS	NS	NS	–	0.002	0.05	NS	NS
28	0.015	–	NS	NS	NS	0.002	–	0.05	NS	NS	0.002	–	NS	NS	NS
120	NS	NS	–	NS	NS	NS	0.05	–	NS	NS	0.05	NS	–	NS	NS
240	NS	NS	NS	–	NS	NS	NS	NS	–	NS	NS	NS	NS	–	NS
365	NS	NS	NS	NS	–	NS	NS	NS	NS	–	NS	NS	NS	NS	–
	Adult-cattle (Booster)														
0	–	0.08	0.015	0.015	0.08	–	0.015	0.002	0.002	0.015	–	0.015	0.002	0.002	0.015
28	0.08	–	NS	NS	NS	0.015	–	NS	NS	NS	0.015	–	NS	NS	NS
120	0.015	NS	–	NS	NS	0.002	NS	–	NS	NS	0.002	NS	–	NS	NS
240	0.015	NS	NS	–	NS	0.002	NS	NS	–	NS	0.002	NS	NS	–	NS
365	0.08	NS	NS	NS	–	0.015	NS	NS	NS	–	0.015	NS	NS	NS	–
	Adult-cattle (Non-booster)														
0	–	NS	NS	NS	NS	–	0.05	NS	NS	NS	–	NS	NS	NS	NS
28	NS	–	NS	NS	NS	0.05	–	NS	NS	NS	NS	–	NS	NS	NS
120	NS	NS	–	NS	NS	NS	NS	–	NS	NS	NS	NS	–	NS	NS
240	NS	NS	NS	–	NS	NS	NS	NS	–	NS	NS	NS	NS	–	NS
365	NS	NS	NS	NS	–	NS	NS	NS	NS	–	NS	NS	NS	NS	–

NS: Value non-significant at 5% level of significance.

Anti-SP Abs titer [≥1.65 log10] against FMDV serotype A virus positive proportion over time for different groups of animals immunized with FMD-HS-BQ combined vaccine depicted in Figure 3. The adult-cattle (B) group again shows the most robust response with a peak at 28 DPV that remains high (close to 1.0) until 240 DPV. It differs significantly, before slightly declining by 365 DPV, making it the top-performing group (Table 2). The adult-cattle (NB) group peaks similarly at 28 DPV but then declines steadily, particularly after 120 DPV, indicating a less sustained response compared to adult-cattle (B). The calf-naive (B) group shows a notable increase, peaking at 120 DPV and then maintaining a steady level, although at a lower proportion than the adult groups. The calf naive (NB) group exhibits a strong initial response but rapidly declines after 28 DPV, indicating weak long-term responses. The Control group remains at zero, indicating no positive response and no evidence of FMD virus circulation.

Anti-SP Abs titer [≥1.65 log10] against FMD serotype Asia1 virus positive proportion over time for different groups of animals immunized with FMD-HS-BQ combined vaccine depicted in Figure 3. The adult-cattle (B) group shows the highest and most sustained positive proportion, peaking at 28 DPV and maintaining nearly the same level until 365 DPV, making it the best performer in the context of Asia1 positivity. The adult-cattle (NB) group also peaks strongly at 28 DPV but shows a significant decline over time, particularly after 120 DPV. The calf-naive (B) group increases steadily, reaching its peak at 120 DPV, then remains stable at a high level, making it the second-best performing group. The calf-naive (NB) group exhibits a rapid rise to its peak at 28 DPV but declines sharply after 120 DPV, indicating a weaker long-term response. The control group showed no positive response throughout the period, as expected.

### 3.2 Confirmation of protective antibody titer against FMD virus at 365 days post-vaccination by virus neutralization test

SPC ELISA was applied for the estimation of protective Ab titer (Ab titer ≥ 1.65 log_10_ is considered protective and Ab titer < 1.65 log_10_ is unprotected) of FMDV serotypes O, A, and Asia1 in cattle at different points of time, as per the WOAH guideline for PVM. VNT was used to estimate and confirm the presence of neutralizing Abs against different serotypes of FMDV in serum samples collected on 365 DPV. More than 80% of adult-cattle (B) group showed protective Ab titer (≥1.65 log_10_) against all three serotypes of FMDV till 365 DPV. Similarly, in the calf naïve (B) group, more than 80% showed protective Ab titer (≥1.65 log_10_) against serotype O and Asia1, whereas only 50% population showed protective Ab titer (≥1.65 log_10_) against serotype A after 365 DPV (Figure 4).

### 3.3 Polynomial regression analysis for anti-structural protein antibody against FMDV

The polynomial regression model was used to predicts the persistence of protective antibody titer for each FMDV serotype (O, A, and Asia1) at later time points (370 and 400) beyond the range of direct observation for different groups [(calf-naïve (B), adult-cattle (B), calf naïve (NB), adult-cattle (NB), and control)] as depicted by the triangles on the graph (Figure 5). The control group remains at a constant value of 1.50 at all-time points, as expected, due to lack of a specific antibody response against FMDV serotype O, A and Asia 1 but booster and non-booster group have different value at different time point (Figure 5).

In FMDV serotype O, the anti-SP Ab kinetics show that adult-cattle (B) and calf-naïve (B) have more vigorous and longer-lasting immune responses than their non-boosted (NB) equivalents. The adult-cattle (B) group demonstrates the strongest seroconversion, with antibodies increasing from a 0 DPV baseline of 1.65 to a peak of 2.40 at 120 DPV, with a slow decline thereafter but still above baseline (1.69 at 400 DPV), demonstrating effective and long-lasting immunogenicity. The calf-naïve (B) group, with a lower overall peak (1.97 at 120 DPV), shows consistent antibody levels across time points, indicating a consistent immune response even in younger animals. Conversely, the NB groups have an initial peak, most significantly in adult-cattle (NB), with a value of 2.28 at 28 DPV, but then drop more steeply, reaching 1.29 at 400 DPV. The calf-naive (NB) group has a steep fall from its initial peak, suggesting a less prolonged response.

For FMDV serotype A, the temporal trends in anti-SP Ab levels reflect enhanced immunogenicity of the groups, specifically the adult-cattle (B) group. The adult-cattle (B) group demonstrates a significant rise in antibody from baseline (1.50) to a peak of 2.20 on 120 DPV. Though a gradual decline follows, antibody levels remain above baseline throughout the study, reaching 1.58 on 400 DPV, indicating an indurated and persistent antibody response. The calf-naïve (B) group also demonstrates a steady buildup to 1.90 by 240 DPV, with relatively stable titer through to 400 DPV (1.70), reflecting sustained serological activity despite a lower overall peak. By contrast, the non-boosted groups (NB) demonstrate a more fleeting response. The adult-cattle (NB) group peaks at 2.28 on 28 DPV, then declines significantly, with titer falling to 1.44 by 400 DPV. Likewise, the naïve (NB) group demonstrates an initial increase (2.18 on 28 DPV) but declines rapidly to baseline levels by 240 DPV and remains low to the end of the study.

In FMDV serotype Asia1, the anti-SP Ab response profiles unequivocally demonstrate increased immunogenicity across groups, especially the adult-cattle (B) cohort. The adult-cattle (B) group shows the highest level of antibody boost, from a baseline of 1.50 to an endpoint of 2.27 at 120 DPV. While the titer steadily decreases to 1.48 by 400 DPV, it remains above baseline, showing a sustained humoral response. The calf-naïve (B) group shows a more gradual but consistent rise in antibody titer, from 1.50 at 0 DPV to 1.95 at 365 DPV, with only a slight decrease thereafter, reflecting an ongoing and consistent response even in younger animals. This contrasts with the two non-boosted (NB) groups, which have maintained an early peak followed by a sharp drop. The naïve (NB) group reaches 2.13 on 28 DPV but drops back to baseline at 240 DPV and to 1.37 by 400 DPV, with the adult (NB) group similarly reaching 2.12 before falling to 1.39.

Polynomial regression demonstrated overall good model performance in describing longitudinal antibody dynamics across serotypes, with distinct differences between boosted and non-boosted groups (Table 3). The best fits were obtained for booster-vaccinated naïve calves, with R^2^ values ranging from 0.85 to 0.95, and adjusted R^2^ values of 0.69–0.90 with minimal residual error, confirming that antibody trajectories in this group were highly deterministic, smooth, and accurately represented by a quadratic polynomial model. These results indicate that primary vaccination followed by a booster elicits a uniform and predictable serological response, making polynomial regression an appropriate and effective model for forecasting antibody persistence in immunologically naïve cattle. Model performance in booster-vaccinated adults was moderate but acceptable, reflecting R^2^ values of approximately 0.50–0.70 and larger residual variability. This pattern is biologically consistent with heterogeneous immune memory and pre-existing antibody exposure, which introduces natural variability even when booster vaccination is administered. The weakest fits were observed in non-boosted animals, where negative adjusted R^2^ and higher residual error indicated that immune waning did not follow a smooth parametric decline. Rather than indicating a model deficiency, this outcome reflects inherently irregular, stochastic antibody decay in the absence of boosting, influenced by age, immune history, environmental exposure, and management conditions. Control groups showed static antibody levels with near-zero residual error, validating the regression performance under constant conditions.

**Table 3:** Polynomial regression model fit statistics.

FMD virus	Group	R2	AdjR2	Residual_SE
Serotype O	Calf-Naive (Booster)	0.8473	0.6946	0.1023
Serotype O	Adult-Cattle (Booster)	0.5017	0.0033	0.2881
Serotype O	Calf-Naive (Non-booster)	0.3556	−0.2889	0.3373
Serotype O	Adult-Cattle (Non-booster)	0.5608	0.1215	0.2905
Serotype O	Control	0.5163	0.0326	0
Serotype A	Calf-Naive (Booster)	0.9498	0.8996	0.0555
Serotype A	Adult-Cattle (Booster)	0.6998	0.3997	0.2034
Serotype A	Calf-Naive (Non-booster)	0.2353	−0.5294	0.3652
Serotype A	Adult-Cattle (Non-booster)	0.4891	−0.0218	0.3423
Serotype A	Control	0.5163	0.0326	0
Serotype Asia1	Calf-Naive (Booster)	0.9192	0.8385	0.0716
Serotype Asia1	Adult-Cattle (Booster)	0.5675	0.1351	0.299
Serotype Asia1	Calf-Naive (Non-booster)	0.2745	−0.451	0.3352
Serotype Asia1	Adult-Cattle (Non-booster)	0.3707	−0.2585	0.3178
Serotype Asia1	Control	0.5163	0.0326	0

FMD virus: Foot and mouth disease virus.

## 4 Discussion

Strong immune response and protective antibody titer persistence for a long time are important parameters for the control of any disease (Parida, 2009). In endemic settings like India, where FMD, HS, and BQ diseases are prevalent and prophylactic vaccination is practically the only tool available for prevention, control, and eradication of diseases. For all these reasons, the present study was undertaken to estimate the kinetics of FMDV Abs longitudinally after inoculation of an FMD-HS-BQ combined vaccine in cattle at WLI. The main observation that emerged from this study was that FMDV antibodies persisted up to 365 DPV after inoculation of the FMD-HS-BQ combined vaccine in cattle in a considerable proportion of animals in field conditions.

After deep intramuscular inoculation of the FMD-HS-BQ combined vaccine, no adverse reaction (either systemic or local) was recorded in the present study, like previous reports (Saseendranath et al., 2009). This combo vaccine would benefit animals by enhancing the protective immunity against multiple pathogens with just a single immunization (Muenthaisong et al., 2021). This might explain the absence of new cases of FMD, HS, and BQ in vaccinated animals in the present study. This could be due to the formation and persistence of neutralizing antibodies in animals after inoculation of the FMD-HS-BQ combined vaccine (Rao et al., 1993). Some studies reported that combined immunogens may interfere with the development of immune responses (Vidor, 2007; Oh et al., 2012). When animals were vaccinated with two live virus vaccines simultaneously, the Ab titer against FMDV was lower than normal (Castañeda et al., 1976). Conversely, other studies showed that the combined vaccine had no interference between the two immunogens and produced a prolonged and stable immune response against FMD as demonstrated in the present study (Chhabra et al., 2004; Altaf et al., 2012; Muenthaisong et al., 2021; Prasad et al., 2019). In the present study, protective Ab titer (≥1.65 log_10_) against FMDV has been recorded up to 365 DPV or predicted to be more than 365 DPV. This could indicate that combined immunogens for FMDV are non-interfering, as reported by previous workers (Altaf et al., 2012; Muenthaisong et al., 2021; Prasad et al., 2019). Furthermore, we assumed that the bacterial antigen components present in the combined vaccine may have acted as adjuvants for the FMD component. This hypothesis needs to be established through further studies.

The immune response against FMDV was higher in Gr 2 (adult-cattle) than in Gr 1 (calves), and the differences in Ab titer could be due to anamnestic response, which was observed in multiple-vaccinated animals (Patil et al., 2014; Puentes et al., 2016). The impact of a booster dose of vaccine was observed in the present study. In Gr I (calf, B), 33% animals showed protective Ab titer against FMDV, whereas after booster administration, it increased upto 83%, and the remaining 17% of animals were unprotected up to 365 DPV. Similarly, in Gr 2 (adult-cattle, B), 83% of animals showed protective Ab titers, whereas after booster administration, the percentage reached 100%. The higher and sustained immune response in adult animals as compared to calves may be due to the prior exposure/sensitization with FMDV in the environment or multiple/repeated vaccination. On the other hand, few calves in primo vaccination group (Gr 1) were found unprotected, even after booster dose of vaccination, and it could be due to non-responders against FMD vaccine or poorly developed immune system because their immune system gradually matures with immune cells like B- and T- cell repertoire, accompanied by a diminishment of the naïve cell pool, whereas the memory and terminally developed T effectors cells of limited diversity proliferate. A reduced immune response in calves could also be due to maternal antibodies (Nicholla et al., 1984).

In the present study, protective Ab titer against FMDV was estimated sequentially up to 365 DPV. Protection against FMDV in susceptible populations is determined by estimating neutralizing Abs after vaccination. Vaccinated animals develop humoral Ab responses, which are thought to be the most important determinant of protection against FMDV infection. The FMD vaccine primarily induces a humoral response by various mechanisms. VP1 and VP3 capsid proteins of FMDV act as TLR 2 agonists (Liang and Chang, 2014; Ranjan et al., 2016) and are responsible for the upregulation of interleukin-6 (IL-6). Activated IL-6 stimulates the secretion of IL-21 from CD4pT cells, and IL-21 promotes antibody production by B-cells (Dienz et al., 2009; Parida, 2009). Furthermore, follicular helper T (FTh) cells may also be activated by vaccination and responsible for secretion of IL-21 by lymphoid organ follicles (Breitfeld et al., 2000) and ultimately, IL-21 promotes Ab production by B-cells (Ranjan et al., 2025b). Therefore, in the present study, immuneresponses against all three FMDV serotypes were recorded in cattle after inoculation of the FMD-HS-BQ combined vaccine because FMDV was one component. Additionally, new assays like gamma interferon-based cell-mediated immune response assay, and isotype/avidity-specific ELISAs may help to improve the accuracy of prediction of likely protection in the FMD-vaccinated animals (Oh et al., 2012; Cardoso et al., 2024). However, these tests are not yet to be calibrated on wide range of potency test results and also not available for routine use in post-vaccination monitoring (Kerfua et al., 2024).

The persistence of neutralizing Ab against FMDV was also confirmed on 365 DPV by the VNT, as it is a gold standard test. Although VNT is considered as the gold standard test for estimation of neutralizing Abs, it is cumbersome in nature, when applied to large number of samples for vaccine field efficacy studies. A previous report showed that a significant positive correlation between protection and SPCE and suggested that SPCE might be used in assessiing the immunogenecity of vaccines in place of VNT (Paiba et al., 2004; Banda et al., 2023). Moreover, the sensitivity of the SPCE as shown to be less affected by the choice of viurs strain used in the test (Paiba et al., 2004; Banda et al., 2023). Therefore, to confirm the results of SPCE for the determination of protective as well as neutralizing antibody response at 365 DPV, VNT was conducted on the same set of serum samples (as tested by SPCE). From our analyses, a good correlation was observed between these two assays for the detection of protective antibody response. The WOAH terrestrial manual recommends that such serological assays can be used as an alternative to post-vaccination seromonitoring if the cutoff is validated, considering results from VNT and *in vivo* potency studies. It could safely be presumed that a strong anti-FMD antibody response was induced by this combined vaccine since because persistence of neutralizing antibodies above threshold was demonstrated in a considerable proportion of vaccinated animals even after a year of vaccination. The protective Ab titer against FMDV up to 365 DPV or predicted more in animals of Gr 1 and 2, which received a booster dose. However, in the non-booster group the protective Ab against FMDV was started declining after 120 DPV, i.e., 6 months earlier than booster group. The results of the non-booster group of the current study are consistent with an earlier finding (Massicame, 2012). Possibly the magnitude and durability of the induced antibody response could be a result of individual animal variation, which could be compensated for the field vaccination campaign by timely repeat vaccination at 6-months intervals. It seems that to ensure the persistence of protective Ab titer against FMDV, a booster dose 28 days after primary vaccination, followed by biannual vaccination at 6-monthly intervals, is required (Parida, 2009). Failing which, animals may become vulnerable to FMDV infection about 120 DPV.

No antagonistic effect of other immunogens of the combined vaccine, like HS or BQ, on the induction of immune response against FMD has been recorded in the current study, and similar findings have been reported by previous researchers (Srinivasan et al., 2001; Chhabra et al., 2004; El-Bagoury et al., 2014; Gamal et al., 2014; Kasem et al., 2017). The synergistic effect of different immunogens present in a combined vaccine with FMDV has also been reported by other researchers (Chhabra et al., 2004; Trotta et al., 2015; Liu et al., 2019), which could be one of the reasons for the increased duration of immunity in cattle observed in the present study after booster immunization.

Before implementing the combined FMD-HS-BQ vaccination, antibody titers against HS and BQ should also be monitored. However, based on evidence from current studies, Pasteurella and Clostridium immunogens could be combined with the FMD vaccine to protect against FMD, HS, and BQ without antigenic competition, as no active cases were observed during the study period. The results obtained from other studies on the comparison between the monovac and combined vaccine suggested that the researchers could not find any significant difference between the mean antibody titer against FMDV in combined (FMD + HS) and monovac vaccinated groups in Cattle and small ruminants (Reddy et al., 1997; Kumar et al., 2020). Similarly, in studies carried out using a simultaneous vaccine with other antigens, no significant difference was observed (Joseph and Hedger, 1984; Hedger et al., 1986). However, Chhabra et al. (2004) found the combined vaccine (FMD + HS) to be better than the monovac vaccine in buffalo calves and also showed there was no antigenic competition in the combined vaccine so as to compromise immune response against FMD. Therefore, the FMD-HS-BQ combined vaccine could be implemented at WLI to form an immune belt against these three (FMD, HS, and BQ) major livestock and wildlife diseases, and also to save the vaccination cost, reduce the unnecessary burden on animals and animal keepers. This could also prevent the spillage of pathogens either from domestic livestock to wildlife or from wildlife to the domestic livestock population due to the creation of an immune belt at the WLI. Such combination will necessarily require strict vaccine quality control (QC) for the component, which is targeted for disease elimination, i.e., FMD in case of India. This is in line with the progressive control pathway, where other large ruminant diseases are required to be tackled with major diseases, i.e., FMD, and also major small ruminant diseases are to be tackled only with other diseases, i.e., peste des petits ruminants (PPR) with sheep pox vaccine (SPV) and goat pox vaccine (GPV), etc. Due to the long lifespan of large ruminants, it is advisable to carry out studies on duration of immunity in repeatedly vaccinated cattle in the large numbers to asses critical duration of persistence immune response to decide frequency of vaccination using such a combined and quality control (QC) passed vaccination.

In this study, we used the serum samples from the cattle populations that have been vaccinated with the combined vaccine at the WLI area, and used the serum samples for the detection of anti-FMDV SP antibody response. However, there are limitations in our study, and these are listed. In this study, we do not have immunological data from buffalo and small ruminants that share the same grazing area. A significant limitation of this study is that post-vaccination antibody levels against HS and BQ were not evaluated; therefore, the actual immune status and vaccine efficacy in the surveyed animals could not be confirmed. Furthermore, we do not have the epidemiological data on the relative contribution of different livestock species to FMD, HS, and BQ transmission at the study site. Therefore, the findings of our study are entirely cattle-specific and may not be extrapolated to the entire multi-host system at the WLI.

## 5 Conclusion

In the absence of a controlled comparison with monovalent FMD vaccines, this study demonstrates that the FMD-HS-BQ combined vaccine elicits FMD antibody responses that persist for up to 365 days with booster vaccination in cattle. From the present study, it can be concluded that combined vaccines could play an important role in the prevention and control of FMD, HS, and BQ diseases; however, further studies are required to confirm the field-protective efficacy and to evaluate immune responses to HS and BQ components before recommending inclusion of combined vaccines in the vaccination schedule. Although a combined vaccine may be used at the wildlife-livestock interfaces to form an immune belt against these three (FMD, HS, and BQ) major diseases and also to save the vaccination cost, further detailed studies are required to determine the field-protection and efficacy data of the combined vaccine on different livestock species sharing the common grazing areas.

## Data Availability

The original contributions presented in the study are included in the article/Supplementary material, further inquiries can be directed to the corresponding author.
