# Supplementary material for: Kinetics of foot-and-mouth disease antibody response in cattle vaccinated with FMD-HS-BQ combined vaccine under field condition
Source: Front Microbiol. 2026 May 22;17:1800886. doi: 10.3389/fmicb.2026.1800886 (PMC13236550; doi:10.3389/fmicb.2026.1800886)
Supplement: Supplementary file 1 [file Table_1.doc]

**Table S1.** Number of animals showing anti-structural protein antibody titre, ≥1.65 (log_10_) for foot-and-mouth disease virus (FMDV) serotype O, A, Asia1 against FMD virus measured by solid phase competitive ELISA (SPCE) in non-descript Indigenous cattle after inoculation of FMD-HS-BQ combined vaccine at a different time interval.

| **Sl. No.** | **Animal Id** | **Anti-SP antibody titre against FMDV, ≥1.65 (log) at 0 days** | | | **Anti-SP antibody titre against FMDV, ≥1.65 (log) at 28 days** | | | **Anti-SP antibody titre against FMDV, ≥1.65 (log) at 120 days** | | | **Anti-SP antibody titre against FMDV, ≥1.65 (log) at 240 days** | | | **Anti-SP antibody titre against FMDV, ≥1.65 (log) at 365 days** | | |
| --- | --- | --- | --- | --- | --- | --- | --- | --- | --- | --- | --- | --- | --- | --- | --- | --- |
|  |  | **Serotype ‘O’** | **Serotype ‘A’** | **Serotype ‘Asia 1’** | **Serotype ‘O’** | **Serotype ‘A’** | **Serotype ‘Asia 1’** | **Serotype ‘O’** | **Serotype ‘A’** | **Serotype ‘Asia 1’** | **Serotype ‘O’** | **Serotype ‘A’** | **Serotype ‘Asia 1’** | **Serotype ‘O’** | **Serotype ‘A’** | **Serotype ‘Asia 1’** |
|  | **a. Calf- naïve (Booster)** | | | | | | | | | | | | | | | |
| 1 | WL-S-1420 | <1.5 | <1.5 | <1.5 | **1.95** | <1.5 | **1.95** | **1.95** | **1.95** | **1.65** | 1.50 | **1.80** | **1.80** | 1.35 | 1.35 | **1.8** |
| 2 | WL-S-1422 | <1.5 | <1.5 | <1.5 | <1.5 | <1.5 | <1.5 | **2.25** | **1.95** | **2.25** | **>2.1** | **>2.1** | **>2.1** | **>2.1** | **2.1** | **>2.1** |
| 3 | WL-S-1423 | <1.5 | <1.5 | <1.5 | **>2.4** | <1.5 | **2.25** | **2.25** | **1.65** | **1.65** | **2.1** | **1.8** | **1.8** | **>2.1** | **1.8** | **2.1** |
| 4 | WL-S-1427 | <1.5 | <1.5 | <1.5 | <1.5 | <1.5 | <1.5 | <1.5 | <1.5 | <1.5 | <1.5 | <1.5 | <1.5 | 1.35 | 1.35 | 1.35 |
| 5 | WL-S-1152 | <1.5 | <1.5 | <1.5 | <1.5 | <1.5 | <1.5 | **1.95** | **1.65** | **1.95** | **>2.1** | **>2.1** | **>2.1** | **2.1** | **1.95** | **>2.1** |
| 6 | WL-S-1153 | <1.5 | <1.5 | <1.5 | <1.5 | <1.5 | <1.5 | **1.95** | **1.65** | **1.65** | **>2.1** | **>2.1** | **>2.1** | **>2.1** | **>2.1** | **>2.1** |
|  | **b. Calf- naïve (Non-booster)** | | | | | | | | | | | | | | | |
| 1 | WL-S-1228 | <1.5 | <1.5 | <1.5 | **>2.4** | **2.25** | **>2.1** | <1.5 | <1.5 | <1.5 | <1.5 | <1.5 | <1.5 | <1.5 | <1.5 | <1.5 |
| 2 | WL-S-1229 | <1.5 | <1.5 | <1.5 | **>2.4** | **>2.4** | **>2.4** | **1.65** | <1.5 | **1.8** | <1.5 | <1.5 | <1.5 | <1.5 | <1.5 | <1.5 |
| 3 | WL-S-1230 | <1.5 | <1.5 | <1.5 | **>2.4** | **>2.4** | **>2.4** | **1.8** | **1.65** | **1.95** | <1.5 | <1.5 | <1.5 | <1.5 | <1.5 | <1.5 |
| 4 | WL-S-1224 | <1.5 | <1.5 | <1.5 | **1.95** | **1.65** | **1.95** | **1.65** | 1.15 | **1.65** | <1.5 | <1.5 | <1.5 | <1.5 | <1.5 | <1.5 |
| 5 | WL-S-1237 | <1.5 | <1.5 | <1.5 | **1.65** | **1.95** | **1.65** | **1.95** | **1.95** | **1.8** | <1.5 | <1.5 | <1.5 | <1.5 | <1.5 | <1.5 |
| 6 | WL-S-1235 | **<1.95** | <1.5 | <1.5 | **>2.4** | **>2.4** | **2.25** | **2.25** | **1.95** | **1.95** | <1.5 | <1.5 | <1.5 | <1.5 | <1.5 | <1.5 |
|  | **c. Adult- without a known history of vaccination/infection (Booster)** | | | | | | | | | | | | | | | |
| 1 | WL-S-1424 | **1.95** | <1.5 | <1.5 | **>2.4** | **>2.4** | **>2.4** | **>2.4** | **>2.4** | **>2.4** | **>2.1** | **>2.1** | **>2.1** | **>2.1** | **>2.1** | **>2.1** |
| 2 | WL-S-1429 | **1.65** | <1.5 | <1.5 | **>2.4** | **1.65** | **2.25** | **>2.4** | **2.25** | **1.95** | **>2.1** | **>2.1** | **>2.1** | **>2.1** | **1.95** | **1.95** |
| 3 | WL-S-1430 | <1.5 | <1.5 | <1.5 | **>2.4** | **>2.4** | **>2.4** | **>2.4** | **>2.4** | **>2.4** | **>2.1** | **>2.1** | **>2.1** | <1.5 | <1.5 | 1.5 |
| 4 | WL-S-1433 | **1.65** | <1.5 | <1.5 | <1.5 | <1.5 | <1.5 | **>2.4** | **>2.1** | **>2.4** | **>2.1** | **1.95** | **>2.1** | **>2.1** | **1.8** | **2.1** |
| 5 | WL-S-1434 | **1.65** | <1.5 | <1.5 | **>2.4** | **1.65** | **2.25** | **>2.4** | **1.95** | **>2.4** | **>2.1** | **1.95** | **1.95** | **>2.1** | **2.1** | **>2.1** |
| 6 | WL-S-1435 | <1.5 | <1.5 | <1.5 | **>2.4** | **2.25** | **>2.4** | **>2.4** | **>2.1** | **>2.1** | **>2.1** | **1.95** | **2.1** | **>2.1** | **1.8** | **2.1** |
|  | **d. Adult- without a known history of vaccination/infection (Non-booster)** | | | | | | | | | | | | | | | |
| 1 | WL-S-1234 | **1.65** | <1.5 | <1.5 | **>2.4** | **>2.4** | **>2.4** | **2.25** | **1.95** | **1.95** | **1.8** | <1.5 | <1.5 | <1.5 | <1.5 | <1.5 |
| 2 | WL-S-1197 | **1.65** | **1.65** | <1.5 | **1.65** | **1.65** | <1.15 | <1.5 | <1.5 | <1.5 | <1.5 | <1.5 | <1.5 | <1.5 | <1.5 | <1.5 |
| 3 | WL-S-1200 | **>2.4** | **1.65** | **1.95** | **>2.4** | **>2.4** | **>2.4** | **2.25** | **1.95** | **1.95** | **1.65** | 1.5 | 1.5 | <1.5 | <1.5 | <1.5 |
| 4 | WL-S-1231 | <1.5 | <1.5 | <1.5 | **>2.4** | **>2.4** | **>2.4** | **2.25** | **1.8** | **1.8** | **1.95** | **1.65** | **1.65** | <1.5 | <1.5 | <1.5 |
| 5 | WL-S-1239 | <1.5 | <1.5 | <1.5 | **>2.4** | **>2.4** | **>2.4** | **>2.4** | **2.1** | **>2.4** | **1.95** | 1.5 | 1.5 | <1.5 | <1.5 | <1.5 |
| 6 | WL-S-1240 | <1.5 | **>2.4** | <1.5 | **>2.4** | **>2.4** | **1.95** | **1.65** | <1.5 | <1.5 | <1.5 | <1.5 | <1.5 | <1.5 | <1.5 | <1.5 |
|  | **e. Calf- naïve (control- unvaccinated)** | | | | | | | | | | | | | | | |
| 1 | WL-S-1527 | <1.5 | <1.5 | <1.5 | <1.5 | <1.5 | <1.5 | <1.5 | <1.5 | <1.5 | <1.5 | <1.5 | <1.5 | <1.5 | <1.5 | <1.5 |
| 2 | WL-S-1528 | <1.5 | <1.5 | <1.5 | <1.5 | <1.5 | <1.5 | <1.5 | <1.5 | <1.5 | <1.5 | <1.5 | <1.5 | <1.5 | <1.5 | <1.5 |
| 3 | WL-S-1529 | <1.5 | <1.5 | <1.5 | <1.5 | <1.5 | <1.5 | <1.5 | <1.5 | <1.5 | <1.5 | <1.5 | <1.5 | <1.5 | <1.5 | <1.5 |
| 4 | WL-S-1530 | <1.5 | <1.5 | <1.5 | <1.5 | <1.5 | <1.5 | <1.5 | <1.5 | <1.5 | <1.5 | <1.5 | <1.5 | <1.5 | <1.5 | <1.5 |
| 5 | WL-S-1531 | <1.5 | <1.5 | <1.5 | <1.5 | <1.5 | <1.5 | <1.5 | <1.5 | <1.5 | <1.5 | <1.5 | <1.5 | <1.5 | <1.5 | <1.5 |
| 6 | WL-S-1532 | <1.5 | <1.5 | <1.5 | <1.5 | <1.5 | <1.5 | <1.5 | <1.5 | <1.5 | <1.5 | <1.5 | <1.5 | <1.5 | <1.5 | <1.5 |

**Table S 2.** Number of animals showing anti-structural protein antibody titre, ≥1.65 (log_10_) against foot-and-mouth disease virus (FMDV) serotype O, A, Asia1 measured by solid phase competitive ELISA (SPCE) and virus neutralization test (VNT) and antibody titre of hemorrhagic septicemia against *Pasteurella multocida* at 365 days post-vaccination (DPV) in non-descript indigenous cattle after inoculation of FMD-HS-BQ combined vaccine at 0^th^ and 28^th^ DVP.

| **Sr. No.** | **Animal ID** | **Antibody titre against foot and mouth disease virus** | | | | | |
| --- | --- | --- | --- | --- | --- | --- | --- |
|  |  | **Serotype 'O'** | | **Serotype 'A'** | | **Serotype 'Asia 1'** | |
|  |  | **SPCE** | **VNT** | **SPCE** | **VNT** | **SPCE** | **VNT** |
|  | **a. Calf- naïve (Booster)** | | | | | | |
| 1 | WL-S-1420 | 1.35 | 1.8 | 1.35 | 1.5 | 1.8 | 1.5 |
| 2 | WL-S-1422 | >2.1 | 2.1 | 2.1 | 1.5 | >2.1 | 1.8 |
| 3 | WL-S-1423 | >2.1 | >2.1 | 1.8 | 1.95 | 2.1 | 1.8 |
| 4 | WL-S-1430 | <1.2 | 1.2 | <1.2 | <1.2 | 1.2 | <1.2 |
| 5 | WL-S-1152 | 2.1 | 1.95 | 1.95 | 1.5 | >2.1 | 1.5 |
| 6 | WL-S-1153 | >2.1 | 2.1 | >2.1 | >2.1 | >2.1 | 1.8 |
|  | **b. Cattle-Adult (Booster)** | | | | | | |
| 1 | WL-S-1424 | >2.1 | >2.1 | >2.1 | >2.1 | >2.1 | >2.1 |
| 2 | WL-S-1427 | 1.35 | <1.2 | 1.35 | 1.35 | 1.35 | 1.35 |
| 3 | WL-S-1429 | >2.1 | 1.95 | 1.95 | 1.65 | 1.95 | 1.65 |
| 4 | WL-S-1433 | >2.1 | >2.1 | 1.8 | 1.8 | 2.1 | 1.8 |
| 5 | WL-S-1434 | >2.1 | >2.2 | 2.1 | 1.8 | >2.1 | 1.95 |
| 6 | WL-S-1435 | >2.1 | 2.1 | 1.8 | 1.5 | 2.1 | 1.95 |
| **For FMD: SPCE Ab titre- < 1.65 log_10_  (Not protected), ≥1.65 log_10_  (Protected)** | | | | | | | |
| **For FMD: VNT- Ab titre- < 1.5 log_10_  (Not protected), ≥1.5 log_10_  (Protected)** | | | | | | | |

**Table S3.** Comparison of confidence interval (95% CI, lower, upper) for titre of anti-structural protein (SP) antibodies against foot-and-mouth disease virus at different time interval (days post vaccination, DPV).

| **Group of animals** | **Anti-structural protein (SP) antibodies log_10_ titre**  **(95% CI, lower, upper)** | | | | |
| --- | --- | --- | --- | --- | --- |
|  | **0 dpv** | **28 dpv** | **120 dpv** | **240 dpv** | **365 dpv** |
| **For calf naïve (Booster)** | | | | | |
| FMDV serotype O | (1.50, 1.50) | (1.42, 2.03) | (1.75, 2.20) | (1.65, 2.15) | (1.54, 2.16) |
| FMDV serotype A | (1.50, 1.50) | (1.50, 1.50) | (1.58, 1.87) | (1.70, 2.10) | (1.50, 2.05) |
| FMDV serotype Asia 1 | (1.50, 1.50) | (1.44, 1.96) | (1.55, 2.00) | (1.70, 2.10) | (1.68, 2.22) |
| **For calf naïve (Non-booster)** | | | | | |
| FMDV serotype O | (1.43, 1.72) | (1.94, 2.46) | (1.61, 1.99) | (1.50, 1.50) | (1.50, 1.50) |
| FMDV serotype A | (1.50, 1.50) | (1.93, 2.42) | (1.39, 1.84) | (1.50, 1.50) | (1.50, 1.50) |
| FMDV serotype Asia 1 | (1.50, 1.50) | (1.89, 2.36) | (1.66, 1.89) | (1.50, 1.50) | (1.50, 1.50) |
| **For adult cattle (Booster)** | | | | | |
| FMDV serotype O | (1.52, 1.78) | (1.96, 2.54) | (2.40, 2.40) | (2.10, 2.10) | (1.66, 2.24) |
| FMDV serotype A | (1.50, 1.50) | (1.64, 2.31) | (2.05, 2.35) | (1.96, 2.09) | (1.56, 2.09) |
| FMDV serotype Asia 1 | (1.50, 1.50) | (1.92, 2.48) | (2.12, 2.43) | (2.03, 2.12) | (1.37, 2.18) |
| **For adult cattle (Non booster)** | | | | | |
| FMDV serotype O | (1.42, 1.98) | (2.03, 2.52) | (1.77, 2.33) | (1.56, 1.89) | (1.50, 1.50) |
| FMDV serotype A | (1.44, 1.96) | (2.03, 2.52) | (1.46, 2.03) | (1.24, 1.58) | (1.50, 1.50) |
| FMDV serotype Asia 1 | (1.43, 1.72) | (1.71, 2.52) | (1.45, 2.13) | (1.24, 1.58) | (1.50, 1.50) |

**Note:** For control group all values are (1.50, 1.50) and all the values are expressed log_10_ scale.

**Table S4**. Post-hoc power analysis.

| **FMD virus Serotype** | **Comparison** | **Mean1** | **Mean2** | **SD1** | **SD2** | **Cohen_d** | **Power** |
| --- | --- | --- | --- | --- | --- | --- | --- |
| O | Calf-naive_B vs Cattle adult_B | 1.85 | 1.95 | 0.102 | 0.288 | -0.463 | 0.112 |
| O | Calf-naive_B vs Calf-naive_NB | 1.85 | 1.5 | 0.102 | 0.337 | 1.404 | 0.593 |
| O | Calf-naive_B vs Cattle-adult_NB | 1.85 | 1.5 | 0.102 | 0.29 | 1.607 | 0.709 |
| O | Cattle-adult_B vs Calf-naive_NB | 1.95 | 1.5 | 0.288 | 0.337 | 1.435 | 0.611 |
| O | Cattle-adult_B vs Cattle-adult_NB | 1.95 | 1.5 | 0.288 | 0.29 | 1.555 | 0.681 |
| O | Calf-naive_NB vs Cattle-adult_NB | 1.5 | 1.5 | 0.337 | 0.29 | 0 | 0.05 |
| A | Calf-naive_B vs Cattle adult_B | 1.77 | 1.82 | 0.056 | 0.203 | -0.335 | 0.082 |
| A | Calf-naive_B vs Calf-naive_NB | 1.77 | 1.5 | 0.056 | 0.365 | 1.034 | 0.367 |
| A | Calf-naive_B vs Cattle-adult_NB | 1.77 | 1.5 | 0.056 | 0.342 | 1.101 | 0.407 |
| A | Cattle-adult_B vs Calf-naive_NB | 1.82 | 1.5 | 0.203 | 0.365 | 1.083 | 0.396 |
| A | Cattle-adult_B vs Cattle-adult_NB | 1.82 | 1.5 | 0.203 | 0.342 | 1.137 | 0.429 |
| A | Calf-naive_NB vs Cattle-adult_NB | 1.5 | 1.5 | 0.365 | 0.342 | 0 | 0.05 |
| Asia1 | Calf-naive_B vs Cattle adult_B | 1.95 | 1.77 | 0.072 | 0.299 | 0.828 | 0.255 |
| Asia1 | Calf-naive_B vs Calf-naive_NB | 1.95 | 1.5 | 0.072 | 0.335 | 1.857 | 0.825 |
| Asia1 | Calf-naive_B vs Cattle-adult_NB | 1.95 | 1.5 | 0.072 | 0.318 | 1.954 | 0.861 |
| Asia1 | Cattle-adult_B vs Calf-naive_NB | 1.77 | 1.5 | 0.299 | 0.335 | 0.85 | 0.266 |
| Asia1 | Cattle-adult_B vs Cattle-adult_NB | 1.77 | 1.5 | 0.299 | 0.318 | 0.875 | 0.279 |
| Asia1 | Calf-naive_NB vs Cattle-adult_NB | 1.5 | 1.5 | 0.335 | 0.318 | 0 | 0.05 |
| NOTE: B-Booster; NB- Non-Booster | | | | | | | |

Post-hoc power analysis confirmed that the study design provided good statistical sensitivity for detecting large booster-associated differences in antibody levels, even with a subgroup size of n = 6. Comparisons involving booster-vaccinated naïve calves consistently exhibited large effect sizes and strong power estimates, particularly for serotype Asia1 (Cohen’s d = 1.857–1.954; Power = 0.825–0.861) and serotype O (d = 1.404–1.607; Power = 0.593–0.709). These values demonstrate that the model could reliably detect biologically meaningful increases in antibody persistence attributable to booster vaccination, validating the robustness of major immunogenicity findings. Power estimates for adult animals and comparisons involving non-boosted subgroups were lower, reflecting smaller serological differences and greater biological variability rather than limitations of the statistical approach. In cases where group means were nearly identical (e.g., Calf-NB vs Adult-NB across all serotypes), effect sizes approached zero and statistical power was minimal, which accurately represents the absence of a true biological difference to detect, not a failure of the analytical design. Overall, the post-hoc power analysis shows that the study was well-powered to detect the strongest and most biologically relevant vaccination effects, while weaker power for minor group differences is consistent with subtle or negligible immunogenic variation. Importantly, these findings support the conclusion that booster vaccination in naïve calves produces strong, predictable, and statistically detectable antibody responses, even in a small sample longitudinal field design.
